# RISING STARS: Mechanistic insights into maternal–fetal cross talk and islet beta-cell development

**DOI:** 10.1530/JOE-23-0069

**Published:** 2023-11-08

**Authors:** Seokwon Jo, Emilyn U Alejandro

**Affiliations:** 1 Department of Integrative Biology & Physiology, University of Minnesota Medical School, Minneapolis, Minnesota, USA

**Keywords:** islet cells, diabetes, placenta, pancreas, pregnancy

## Abstract

The metabolic health trajectory of an individual is shaped as early as prepregnancy, during pregnancy, and lactation period. Both maternal nutrition and metabolic health status are critical factors in the programming of offspring toward an increased propensity to developing type 2 diabetes in adulthood. Pancreatic beta-cells, part of the endocrine islets, which are nutrient-sensitive tissues important for glucose metabolism, are primed early in life (the first 1000 days in humans) with limited plasticity later in life. This suggests the high importance of the developmental window of programming *in utero* and early in life. This review will focus on how changes to the maternal milieu increase offspring’s susceptibility to diabetes through changes in pancreatic beta-cell mass and function and discuss potential mechanisms by which placental-driven nutrient availability, hormones, exosomes, and immune alterations that may impact beta-cell development *in utero*, thereby affecting susceptibility to type 2 diabetes in adulthood.

## Invited Author’s profile


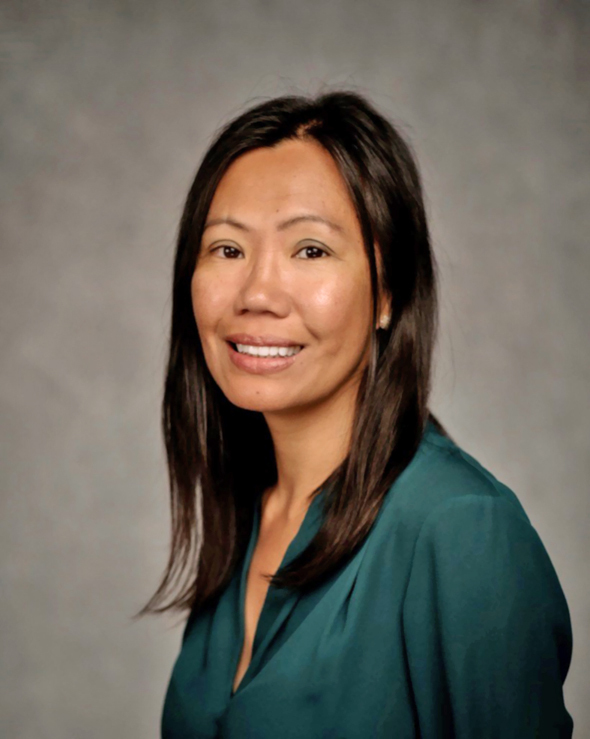


**Dr Emilyn U Alejandro** is an associate professor at the University of Minnesota Medical School’s Department of Integrative Biology and Physiology (IBP). Her academic journey began in 2000 at the University of Washington, where she conducted type 1 diabetes research as an undergraduate McNair Scholar at Dr Ake Lernmark’s lab. She furthered her scientific passion by participating in a postbaccalaureate program at the National Cancer Institute at NIH under Dr Elise Kohn’s guidance. In 2010, Dr Alejandro completed her doctoral training at the University of British Columbia in Canada, under the mentorship of Dr James D Johnson, with support from an NIH F31 grant. She pursued her postdoctoral training at the University of Michigan under the mentorship of Dr Ernesto Bernal-Mizrachi and was generously funded by NIH T32 and K01 Career Development Award. As an independent investigator at the University of Minnesota Medical School, Dr Alejandro’s research focuses on nutrient sensor proteins’ effects in the placenta and pancreas, advancing our understanding of the fetal origins of obesity and type 2 diabetes. Her exceptional work has earned her numerous accolades, including the McKnight Land-Grant Professorship, McKnight Presidential Fellow, and the APS Henry Pickering Bowditch Lectureship Award. Beyond her research, Dr Alejandro is dedicated to mentoring and supporting students from diverse backgrounds. She actively contributes to the scientific community through editorial boards and NIH Study Section membership. Despite her busy career, she cherishes time with her family, finding joy in watching her daughters play ice hockey and swim, as well as traveling around the world. Dr Emilyn Alejandro’s remarkable achievements and commitment to advancing knowledge make her a prominent figure in her field and a source of inspiration for future scientists.

## Introduction

Type 2 diabetes (T2D) is a major health and economic concern worldwide, with its prevalence steadily increasing. Its origins are complex, involving a mix of genetic factors and exposure to environmental elements over a person’s lifetime (Lango *et al*. 2008,Bruce & Byrne 2009). T2D is a multifaceted condition that primarily arises due to the pancreatic beta-cells not expanding and functioning adequately. These beta-cells are responsible for producing and releasing insulin, an anabolic hormone crucial for regulating stable glucose levels by promoting glucose absorption in insulin-sensitive peripheral tissues. During T2D, the shortfall of beta-cells results in the inability to meet the body’s insulin demands, especially in the face of insulin resistance in peripheral tissues such as the skeletal muscle or adipose (Alejandro *et al.* 2014*a*). Research indicates that people with impaired fasting glucose and glucose intolerance tend to have deficiencies in both the overall quantity of beta-cells (known as beta-cell mass) and their performance (Butler *et al.* 2003). While lifestyle choices in adulthood play a significant role in T2D development, additional factors during fetal development also influence the growth and function of beta-cells (Simmons *et al.* 2001), which in turn affects their mass and performance. However, detailed and causal mechanistic investigations in this area are still limited.

The concept of Developmental Origins of Health and Disease (aka DOHaD) suggests that the time during pregnancy, in the womb, and right after birth are crucial periods that significantly influence how the tissues of the developing offspring are programmed. Because of this, if important metabolic tissues like the pancreas are programmed incorrectly during these critical periods, it can potentially lead to lasting impacts on the health trajectory and vulnerability of the offspring to conditions like obesity and T2D in adulthood (Heindel *et al.* 2015, Hoffman *et al.* 2021). For example, prenatal complications such as maternal malnutrition and gestational diabetes are associated with negative metabolic impacts in the offspring, including increased glycemia, weight gain, insulin resistance, and reduced pancreatic beta-cell mass – all critical contributors to the development of T2D (Lesage *et al.* 2004, Mueller & Bale 2006).

During development, beta-cells arise from progenitor cells located in the endoderm of the developing embryo. These progenitor cells differentiate into islet cells, including insulin-producing beta-cells, which comprise the endocrine pancreas. This process is regulated by a complex interplay of genetic and environmental factors, including maternal nutrition levels and stressors such as inflammation. Beta-cell mass is set early in life with limited replication capacity later in adulthood (Dor *et al.* 2004, Gregg *et al.* 2012). In rodent models, the replicative capacity is thought to diminish to about 2% per day in postpuberty adulthood, whereas in humans, the beta-cell replication reaches its lower threshold by 2–3 years of age (Finegood *et al.* 1995, Meier *et al.* 2008, Wang *et al.* 2015). The amount of pancreatic beta-cell mass is determined by the quantity and early developmental programming of pancreatic progenitor cells (Stanger *et al.* 2007). This implies that the conditions experienced in the fetal environment can influence the capacity of these specialized cells to grow, perform their functions, and subsequently respond to metabolic challenges across life span.

The placenta plays a crucial role in regulating fetal metabolism during pregnancy. As an endocrine organ, it acts as a gatekeeper, controlling the transfer of nutrients and other substances between the mother and the developing fetus. Recent research publications have shown that alterations in placental function can have significant impacts on offspring metabolism and long-term health outcomes. For example, placental insufficiency that occurs in preeclampsia, can induce fetal growth restriction (FGR) and an increased risk of metabolic disorders, such as T2D, in the adult offspring (Akhaphong *et al.* 2021*a*, Armengaud *et al.* 2021). Direct manipulation of the placenta to induce FGR has also been shown to increase risk for insulin resistance and obesity (Akhaphong *et al.* 2021*a*). In cases of gestational diabetes, elevated maternal glucose levels pass through the placenta to the fetus. If glucose level is left uncontrolled, this can result in macrosomia, a condition characterized by excessive fetal growth. Furthermore, the placenta is not just a passive conduit for nutrients and other molecules. It is a metabolically active organ that produces a variety of hormones and signaling molecules such as leptin, progesterone, adiponectin, and microRNAs (miRNAs) and more that have not been fully characterized yet (Stern *et al.* 2021, Xu *et al.* 2021). Imbalances in the production or activity of these signaling molecules can lead to metabolic disorders in the offspring (Kusuyama *et al.* 2021, Lopez-Tello *et al.* 2023). The intricate interaction between the placenta and fetal metabolism is a field that has received limited attention, yet it is gaining momentum as an active area of investigation. This research holds significant importance, as it has far-reaching implications for the metabolic health of both mothers and their offspring.

**Figure 1:**
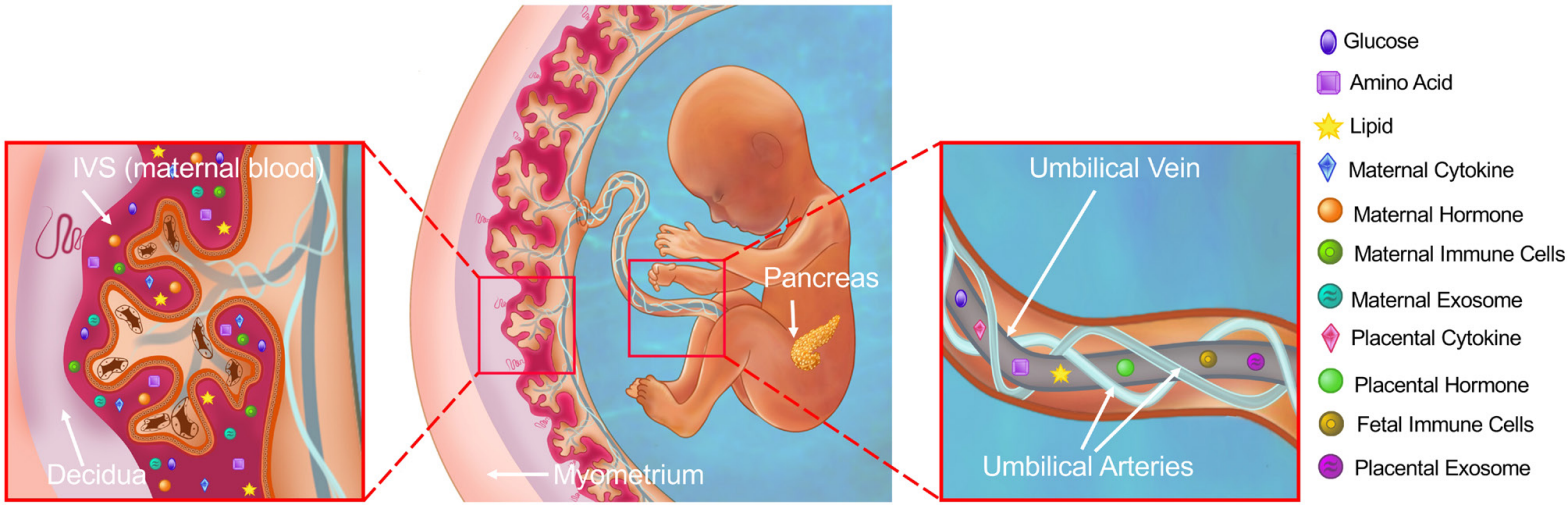
Alterations to maternal health and nutrient milieu leads to differential programming of beta-cell mass and function in the offspring. Placenta is the main interface between mother and fetus that orchestrates the transfer of nutrients, but the placenta also produces secretagogues in response to maternal health that may impact beta-cell development. Possible mechanisms that may impact the total number ( or mass) and function of pancreatic beta-cells include the following: (i) availability of nutrients such as glucose, amino acid, and lipids to beta-cells; (ii) in response to maternal inflammation (e.g. macrophage infiltration and increased maternal cytokines), placenta produces and secretes cytokines that may impact beta-cells directly or indirectly via modulation of the fetal immune system; (iii) placenta produces and releases exosomes which holds contents such as miRNA that may regulate the translational landscape of beta-cells during development; and (iv) placenta is an endocrine organ that secretes hormones such as placental lactogen to fetal circulation that may impact beta-cell growth.

**Figure 2:**
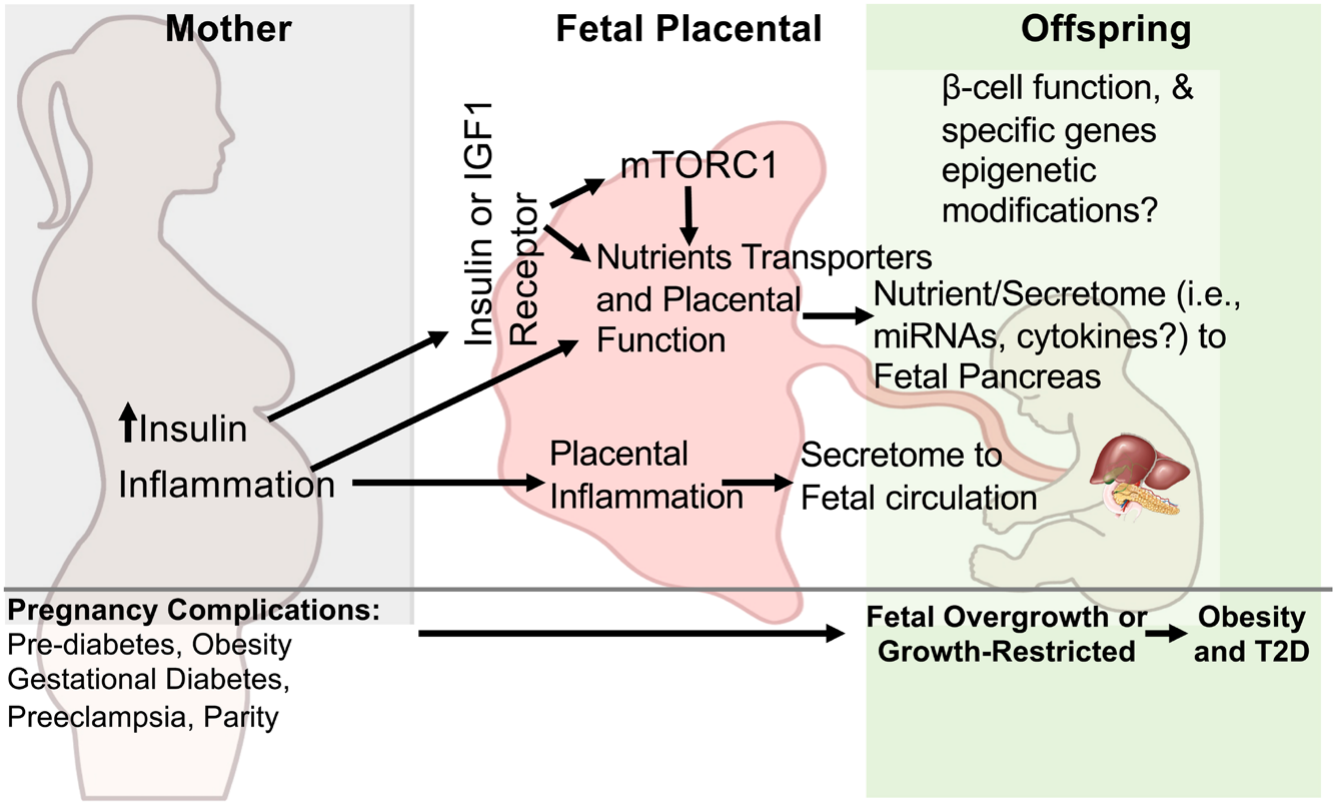
Factors influencing fetal pancreas development and function. The fetal origin of beta-cell dysfunction may stem from alterations in the maternal intrauterine environment, which in turn affects placental function: nutrient transport and secretome content modulation, involving molecules like miRNAs. For instance, the maternal metabolic state, encompassing factors such as hyperinsulinemia, inflammation, preeclampsia, and obesity, brings about modifications in placental development and function during gestation. These alterations can subsequently lead to shifts in nutrient levels and alteration of secretome content into the fetal circulation. These changes hold the potential to influence the programming of various organs such as the reduction of functional beta-cell mass in the offspring.

While the pregnancy period plays a crucial role in influencing fetal growth, there is currently a lack of understanding regarding the molecular mechanisms that link the maternal environment and placental function to the development of fetal pancreatic beta-cells in the offspring. In this review, our objective is to offer an overview of how maternal conditions such as gestational diabetes and malnutrition/overnutrition might influence the metabolic health of the offspring. Additionally, we will highlight mechanisms that could directly affect the development of beta-cells (Fig. 1 and 2). Ultimately, these mechanisms have implications for the susceptibility of the offspring to obesity and T2D.

## Overview of maternal milieu and offspring metabolic health

### Maternal under- and overnutrition

Maternal nutrition during pregnancy has a significant impact on the metabolic health of offspring. Adequate intake of essential nutrients such as glucose, protein, lipid, vitamins, and minerals is crucial for the proper development of the fetus. Studies have shown that maternal nutrient intake during pregnancy can program the metabolic pathways of the fetus and affect the development of key organs such as the pancreas. As early as 2011, the International Federation of Diabetes acknowledged that poor nutrition during pregnancy is a risk factor for developing T2D (https://diabetesatlas.org/atlas/fifth-edition/).

Maternal undernutrition, characterized by a lack of essential nutrients such as amino acids, can result in low birthweight and increased risk of chronic diseases such as obesity, T2D, and cardiovascular disease in later life. Prime examples of human studies include the Dutch and Chinese famine cohort studies, which both demonstrated that severe maternal undernutrition increased the risk of T2D and obesity in the offspring (van Abeelen *et al.* 2012, Yao *et al.* 2023). Placental insufficiency arising from undernutrition may impact offspring health because impairments in placental development (reduced uteroplacental blood flow, improper trophoblast invasion, reduced nutrient and oxygen flux to the fetus, and hormonal imbalances) may affect the intrauterine environment (Zhang *et al.* 2015, Barrientos *et al.* 2017, Vacher *et al.* 2021). The growing pancreatic beta-cells are exquisitely attuned to nutrient status (e.g. glucose, amino acids, and lipids) especially during the developmental period (Guillemain *et al.* 2007, Rachdi *et al.* 2012, Elghazi *et al.* 2017, Baumann *et al.* 2020). This is corroborated by several rodent studies that highlight maternal consumption of a low-protein diet during pregnancy reduces beta-cell mass and function of the offspring, leading to glucose intolerance as adults (Petrik *et al.* 1999, Alejandro *et al.* 2014*b*, 2020, King *et al.* 2019). It is also important to note that total calorie restriction in the dams causes a similar result of beta-cell mass deficit in the offspring (Dumortier *et al.* 2007).

The strong association between abnormal birthweight and risk of adverse metabolic health outcomes is U-shaped. Thus, both children who are born small and those who are born large for gestational age are at risk for the eventual development of obesity, T2D, and cardiovascular disease later in life. As such, maternal overnutrition is also correlated with a poor metabolic health trajectory in the offspring (Ismail-Beigi *et al.* 2006). As the prevalence of obesity at the time of conception and during pregnancy are rapidly rising (Kim *et al.* 2007, Driscoll & Gregory 2020, Wang *et al.* 2021), maternal obesity may be a significant factor that further exacerbates the generational epidemic of obesity. Maternal obesity is correlated with pregnancy complications such as gestational diabetes and preeclampsia (Catalano 2010, Kong *et al.* 2020) and is highlighted as a risk factor for the development of diabetes in the offspring through mal-adaptive programming of nutrient sensitive tissues such as adipose tissue and pancreatic islets (Lecoutre *et al.* 2021). Animal models of a maternal obesogenic diet have demonstrated this link between overnutrition and offspring beta-cell programming. Different models of maternal high-fat diet in rodents report altered islet morphology, beta-cell mass, and function (Han *et al.* 2005, Nicholas *et al.* 2020, Akhaphong *et al.* 2021*b*, Casasnovas *et al.* 2021). However, recapitulating human pathophysiology by modeling maternal obesity through dietary alterations is difficult and varying, due to differences in the composition of diet, length of diet fed prior to pregnancy to mimic obesity, and diet change post birth. Other animal studies in sheep and nonhuman primates also show maternal obesity is associated with altered beta-cell function or mass (Ford *et al.* 2009, Elsakr *et al.* 2019). It is important to note that conditions of hypernutrition, such as maternal obesity, not only comes with increased nutrient availability but also hormonal (e.g. maternal insulin, leptin) and inflammatory (e.g. cytokine IL-8, IL-6, TNF) changes that may impact placental function and offspring metabolic health (Kelly *et al.* 2020, Denizli *et al.* 2022). Extensive reviews on the impact of maternal nutrition on offspring metabolic health are found here (Kunz & King 2007, Caton *et al.* 2019, Odhiambo *et al.* 2020).

### Pregnancy complications that may impact pancreatic beta-cell development

Various pregnancy complications such as fetal growth restriction from preeclampsia or macrosomia from gestational diabetes are increasing, and they contribute to adverse pregnancy outcomes and alterations in offspring metabolic health trajectory (Cameron *et al.* 2022, Zhou *et al.* 2022). While the etiology of these complications is complex, their impact on the placenta and the fetus are also poorly understood. Despite the ample body of evidence that suggests these mal-adaptive *in utero* environments greatly impact the offspring disease susceptibility in adulthood, the mechanisms describing how and when such malprogramming occurs remains scant and warrants further investigation.

FGR is characterized by a low birth weight, which is defined as a weight that is below the 10th percentile for gestational age. This condition can result from a variety of factors, including maternal malnutrition, maternal smoking and preeclampsia (Chew & Verma 2022). Underlying mechanisms linking FGR to increased risk of metabolic disorders are not fully understood, but it is thought that changes in the intrauterine environment, such as reduced oxygen and nutrient availability and exposure to adverse maternal metabolic conditions, may play a role. Epidemiological studies have consistently shown that individuals who were born with FGR have an increased risk of developing insulin resistance, which is a key factor in the development of T2D in adulthood (Van Assche *et al.* 1977, Veening *et al.* 2002). Animal models of FGR support the hypothesis that beta-cell development deficits and insulin secretion defects observed in these models contribute to poor glucose metabolism (Akhaphong *et al.* 2018, Jabary *et al.* 2023). The effects of FGR in beta-cell development is well-documented by several review publications (Boehmer *et al.* 2017, Mohan *et al.* 2018).

As the prevalence of T2D increases, especially in the developing countries (Nanditha *et al.* 2016), gestational diabetes (GDM) is another critical pregnancy complication where the effects of excess glucose, in addition to different impacts on maternal metabolism, such as hyperinsulinemia and obesity, on the fetal programming of metabolic health must be considered. As normal pregnancy proceeds, pancreatic beta-cells of the mother proliferate and secrete insulin to meet the needs for the metabolic alterations that comes with concurrent rise in insulin demand (Powe *et al.* 2019). When this demand is unmet, hyperglycemia develops with detrimental consequences for the mother and the fetus. Observational studies in humans suggest that offspring from mothers who developed GDM have increased weight gain and impaired glucose metabolism (Silverman *et al.* 1991, Pettitt & Knowler 1998, Fetita *et al.* 2006, Dabelea *et al.* 2008, Holder *et al.* 2014). Impaired glucose tolerance observed in offspring of mothers who developed GDM has been associated with reduced beta-cell function (Sobngwi *et al.* 2003, Holder *et al.* 2014, Lowe *et al.* 2019). However, mechanistic insights into changes in beta-cell mass and function from the GDM fetus/offspring are lacking. Some of the early work in rat models of GDM using streptozocin (beta-cell specific apoptosis-inducer) treated dams suggest that GDM offspring had increased islet mass associated with islet hyperplasia, and increased circulating insulin levels neonatally (Kervran *et al.* 1978, Bihoreau *et al.* 1986*a*, Aerts *et al.* 1990). However, later in adulthood, both *in vivo* and *ex vivo* islet insulin secretion were suppressed with concomitant impaired glucose tolerance in the GDM offspring, suggesting beta-cell exhaustion from early beta-cell hyperplasia and function (Bihoreau *et al.* 1986*b*, Aerts *et al.* 1988, Gauguier *et al.* 1991). In other mouse models of GDM, such as diet induced (high fat/high sucrose), islet mass was increased with greater alpha-cell fraction in neonates. When the offspring were further subjected to metabolic stress, these GDM offspring had a reduced insulin secretion index with impaired glucose tolerance (Agarwal *et al.* 2019). Additionally, offspring from dams of transgenic insulin resistance and GDM (liver insulin receptor knockout mice) shows higher circulating insulin levels despite reduced offspring beta-cell area with reduced proliferation and total islet number (Kahraman *et al.* 2014), suggesting complex changes to both insulin producing beta-cells and insulin-sensitive peripheral tissues. GDM is a multifactorial complication and is not fully recapitulated in current animal models used, which leads to varying effects on fetal tissue development and function. While mechanisms as simple as increased glucose availability to the fetus may explain some of the changes in offspring islet development and function, other mechanisms involving placental function and inflammatory status in dams and placenta must be considered.

As a woman ages, her reproductive health and likelihood of conceiving decreases. Such pregnancies are associated with increased risks during pregnancy and at childbirth (Cavazos-Rehg *et al.* 2015). Aging is positively associated with obesity, inflammation, and metabolic disorders – all determinants of adverse offspring health (Jura & Kozak 2016, Chung *et al.* 2019). As such, advanced maternal age is also a risk factor for FGR (Odibo *et al.* 2006) and has been associated with increased offspring obesity (Myrskyla & Fenelon 2012). In addition to aging, parity, defined as number as pregnancies with a gestational age of 24 weeks or more, is associated with adverse maternal health such as inflammation and obesity (Gaillard *et al.* 2013, Ndiaye *et al.* 2018, Dasa *et al.* 2022, Ding *et al.* 2022). In human cohort studies, offspring from multiparous pregnancy have improved cardiometabolic health at young age with less adverse neonatal outcomes, compared to the offspring from nulliparous mothers (Kozuki *et al.* 2013, Gaillard *et al.* 2014). Longitudinal studies are needed as maternal programming-mediated offspring pathophysiology such as obesity, T2D, and cardiovascular diseases themselves are age associated. In a mouse model study of parity, consistent to human studies, multiparous dams were associated with maternal obesity and inflammation independent of maternal age. This was associated with increased obesity in the adult male offspring (Rebholz *et al.* 2012). Additionally, multiparity positively associated with glucose intolerance in nonhuman primates at 3 years of age (Elsakr *et al.* 2019). The negative metabolic effect on offspring from multiparity appears to be correlated with placental insulin signaling, as deletion of placental insulin receptor improved glucose homeostasis in multiparous offspring (Chung *et al.* 2022). Further studies are needed to examine the alterations to islet and beta-cell physiology in the context of maternal aging and parity.

### Maternal inflammation

Alterations to the maternal immune system is a required step in healthy pregnancies to foster normal placental development as well as maintain optimal fetal health. While inflammation is a normal response to infection or injury, acute or chronic low-grade inflammation engaged by viral infection or maternal obesity may lead to maladaptive immunologic changes in both the mother and fetus that negatively impact the placenta and, thus, offspring growth, and development.

Viral infections during pregnancy can have a significant impact on the metabolic health of both the mother and the offspring. Viral infections can impair placental function, causing placental insufficiency resulting in FGR and leading to decreased fetal growth and increased risk of adverse outcomes (Rathore *et al.* 2022). Studies have shown that some viral infections, such as rubella, cytomegalovirus, and Zika virus, can cross the placenta and infect the fetus, leading to increased risk of congenital anomalies and developmental delays (Gordon-Lipkin *et al.* 2021). An *ex vivo* study in human fetal islets indicates that rubella can infect fetal beta-cells and impair insulin secretion, suggesting a potential direct impact of viral infection on fetal beta-cells during pregnancy (Numazaki *et al.* 1989). Furthermore, viral infections during pregnancy can trigger maternal inflammation and oxidative stress, which can have long-term effects on fetal metabolism (Auriti *et al.* 2021). These effects may be due to changes in the expression of genes involved in lipid metabolism in the placenta, leading to altered metabolic pathways and increased risk of metabolic disorders later in life (Chen *et al.* 2020). In recent years, coronavirus disease 2019 (COVID-19), caused by the novel coronavirus severe acute respiratory syndrome coronavirus 2 (SARS-CoV-2), has become a global pandemic, affecting millions of people worldwide. Pregnant women are particularly vulnerable to the effects of COVID-19, and the virus has been shown to have a significant impact on placental and fetal health (Rad *et al.* 2021, Shook *et al.* 2022). owever, not all studies have identified higher percentages of placental pathology or vertical transmission.

Evidence suggests that SARS-CoV-2 RNA is detected in the placenta of infected women, leading to increased immune infiltration as well as increased expression of chemokines and inflammatory markers (Argueta *et al.* 2022). Additionally, in some cases, maternal COVID-19 infection associated with acute respiratory distress syndrome has been linked with altered metabolic response in the fetus due to the lack of oxygen transfer (Jovandaric *et al.* 2022). Sexual dimorphic responses of the placenta to COVID-19 infection with increased impact on male placental and neonatal immunity has been reported (Bordt *et al.* 2021). While it is possible that COVID-19 infection during pregnancy may impact offspring susceptibility to diabetes (Eberle *et al.* 2021), there is limited evidence on how fetal pancreatic islet development is specifically altered in response to COVID-19 infection *in utero*.

Recent studies have highlighted immunometabolism as critical nexus of tissue cross talk with perturbations to this interaction leading to pathophysiological conditions such as diabetes. Obesity is associated with chronic inflammation that may exacerbates the disease progression. Strong correlation between circulating TNF and insulin resistance have been reported (Hotamisligil *et al.* 1993). Systemic inflammation is characterized by high circulating levels of inflammatory cytokines and chemokines as well as immune cells that infiltrate different organs (Weisberg *et al.* 2003). This increased inflammatory status of obesity extends during pregnancy and adversely impacts the intrauterine environment (Segovia *et al.* 2017). Maternal obesity has been generally associated with increased placental and fetal weight, reduced placental efficiency (fetoplacental weight ratio), and increased placental inflammatory cell infiltration (Wallace *et al.* 2012, Bianchi *et al.* 2021). However, maternal obesity in animal models also leads to FGR or an increased rate of SGA (Akhaphong *et al.* 2021*b*). These alterations are associated with local macrophages, Treg lymphocyte infiltration, and increased pro-inflammatory mediators such as IL-6 and TNF (Challier *et al.* 2008, Zhang *et al.* 2018). Increased inflammatory status in the placenta is associated with defects in trophoblast migration, apoptosis, and proliferation as well as vascular remodeling (Islami *et al.* 2003, Stuart *et al.* 2018, St-Germain *et al.* 2020). Mechanistically, RNAseq and metabolomics performed on human placentae from obese pregnancies show increased gene expressions in lipid metabolism, cytokine production, and angiogenesis with a shift in lipid profiles (Saben *et al.* 2014, Fattuoni *et al.* 2018). This may contribute to lipotoxic conditions that maternal obesity presents, which induce placental dysfunction via cross talk between nutrient-based stress, insulin resistance, and immune activation. As such, it is difficult to tease apart the relative contribution that increased inflammation by cytokines and immune infiltration have on maternal obesity-derived placental and fetal dysfunction. Studies in animal models injected with lipopolysaccharides (LPS), a bacterial-derived endotoxin commonly used in immunometabolism studies to mimic similar obesity-induced immune activation, show similar metabolic dysfunction in the offspring as in maternal obesity (Nilsson *et al.* 2001, Wei *et al.* 2007, Cadaret *et al.* 2018). While a correlation between maternal inflammation and fetal glucose metabolism has been identified, there are no studies that directly examine the alterations of pancreatic beta-cell number and function, which are acutely attuned to nutrients received from the placenta as well as alterations to metabolic flux. As discussed below, further studies into characterizing immune system-driven alterations to the placenta as well as specific islet studies are needed to study the impact of *in utero* immune modulation in pancreatic beta-cell development.

## Potential mechanisms of placenta mediated beta-cell development

### Nutrient flux and sensing by placenta

Nutrient availability is one of the key determinants of pancreas development and sets the basis for the offspring’s metabolic parameters early in life. One of the key functions of the placental trophoblasts is to transport nutrients, which impacts both placental and fetal growth. Alterations to placental nutrient flux due to changes in nutrient transporters have been associated with maladaptive offspring growth and metabolic health (Herrera *et al.* 2006, Brett *et al.* 2014, Diaz *et al.* 2014). Amino acids are essential for the development of fetal tissues. During the progression of pregnancy, there is increased amino acid transporter activity, and the levels of amino acids in fetus are higher than that in maternal circulation (Cetin *et al.* 1992, Jansson 2001, Desforges *et al.* 2010). In brief, well-studied amino acid transporters include (i) system A (SNAT), a sodium-dependent amino acid transporter that facilitates the transfer of small neutral amino acids such as alanine, glycine, and serine and (ii) system L (LAT), a sodium-independent amino acid transporter for larger neutral amino acids such as leucine. Various pregnancy complications related to placental insufficiency generally leads to lower SNAT and LAT expression (Glazier *et al.* 1997, Jansson *et al.* 1998, Rosario *et al.* 2011, Ganguly *et al.* 2012, Sferruzzi-Perri & Camm 2016). Of note, branched amino acids such as leucine and isoleucine have been shown to be critical in beta-cell development and proliferation, which may explain the phenotypes of maladaptive development of islet cells in these pregnancy complications (Xu *et al.* 1998, Rachdi *et al.* 2012, Elghazi *et al.* 2017). Consistently, direct fetal leucine infusion leads to increased beta-cell function and size (Boehmer *et al.* 2020), suggesting that *in utero* amino acid concentrations play a great role in setting the basis of beta-cell mass.

Glucose, another major nutrient required for fetal growth, is transported through various isoforms of the glucose transporter (GLUT) expressed in the placenta (Illsley & Baumann 2020). GLUT1 is the primary glucose transporter in the placenta, while other GLUTs (GLUT3, 4, 8, 9, 10, and 12) are thought to play a smaller role in fetal glucose transport (Lager & Powell 2012, Mohan *et al.* 2018). As such, placental GLUT1 is often the isoform whose expression is altered in varying pregnancy complications such as GDM, malnutrition, and obesity without changes to other isoforms (Sferruzzi-Perri & Camm 2016, Illsley & Baumann 2020, Stanirowski *et al.* 2022). Postnatal islet studies suggest that glucose stimulates beta-cell proliferation (Cousin *et al.* 1999, Stamateris *et al.* 2016). However, studies on glucose mediated beta-cell development early in life are limited. Fetal hypoglycemia characterized in sheep FGR models is associated with reduced glucose responsiveness in offspring’s islet insulin secretion, and this defect can be restored with glucose infused euglycemic recovery (Limesand & Hay 2003), suggesting that an appropriate glucose level is, indeed, critical in programming beta-cell function and mass *in utero*.

Lipids also serve critical roles in fetal growth during pregnancy (Koletzko *et al.* 2007). Maternal triglyceride/lipids must be processed and transferred via several placental and endothelial lipases, fatty acid-binding proteins, and translocases (Brett *et al.* 2014). Expression of these placental lipid processing and storage proteins are subject to maladaptive conditions such as obesity and FGR, suggesting that placental plasticity is important in modulating fetal lipid flux (Magnusson *et al.* 2004, Rasool *et al.* 2022). Limited studies show a direct correlation between fetal lipid status and their beta-cell development. In human studies, lipid species such as DHA, linolenic acid, and n-3 long-chain polyunsaturated fatty acids (LC-PUFAs) in fetal circulation were positively correlated with cord plasma proinsulin levels while n-6 LC-PUFAs showed negative correlations (Zhao *et al.* 2014). A study in mice suggests that endocannabinoid PUFAs act on the beta-cell cannabinoid receptor and TRPV1 to regulate fetal islet size and organization (Malenczyk *et al.* 2015).

Placental expression levels of the macronutrient transporters discussed above are regulated by various hormones such as maternal insulin, IGF-1, leptin, and cytokines (Jansson *et al.* 2003, Magnusson-Olsson *et al.* 2006, Jones *et al.* 2009, Roos *et al.* 2009*b*, Mousiolis *et al.* 2012, Baumann *et al.* 2014, Nogues *et al.* 2021, Anam *et al.* 2022). A common downstream intracellular target of these hormonal signals is the nutrient sensor protein kinase, mechanistic target of rapamycin (mTOR) (Maya-Monteiro & Bozza 2008, Dong *et al.* 2019, Nakamura *et al.* 2022). mTOR is one of the master nutrient sensor proteins that integrate extracellular signaling pathways such as insulin and nutrient availability to promote growth and development of the cell through regulation of key protein translation complexes and mitochondrial biogenesis (Roos *et al.* 2009*b*). mTOR signaling is also shown to regulate the expression and localization of both amino acid (Roos *et al.* 2007, 2009*a*,*b*) and glucose transport systems (Buller *et al.* 2008), likely impacting the flux of nutrients from mother to fetus. As such, evidence suggests that dysregulation of placental mTOR signaling in humans is associated with FGR and poor pregnancy outcomes (Roos *et al.* 2007, Sati *et al.* 2016, Dimasuay *et al.* 2017, Beetch & Alejandro 2021, Satterfield *et al.* 2021). A recent study revealed a more direct association between placental mTOR and offspring metabolic health. mTOR deletion in the mouse placenta led to lower placental leucine flux from mother to fetus and an FGR phenotype. These offspring exhibit a poor metabolic health outcome, specifically female mice, which exhibit insufficient beta-cell adaptation under metabolic stress (Akhaphong *et al.* 2021*a*).

mTOR signaling is important for not only placental development but also for maintenance of beta-cell mass and function in the pancreas. Alterations to the flux of amino acids or glucose to fetal circulation may impact mTOR activity in islets *in utero*, thereby impacting beta-cell development. Supporting this, branched chain amino acids such as leucine can activate mTOR with direct impacts on pancreatic progenitor and beta-cell development (Rachdi *et al.* 2012, Elghazi *et al.* 2017). Evidence from direct perturbation of mTOR signaling in mouse beta-cell further corroborates the importance of this pathway, as mTOR deletion leads to diabetes, due to loss of beta-cell mass and function in mice (Blandino-Rosano *et al.* 2017, Yin *et al.* 2020). Temporally, the switch in mTOR signaling early in life is shown to be critical for postnatal beta-cell maturation to achieve fully insulin secretory capacity (Jaafar *et al.* 2019, Helman *et al.* 2020). The reduction of islet mTOR signaling in models of maternal malnutrition is associated with reduced beta-cell mass (Alejandro *et al.* 2014*b*, King *et al.* 2019), suggesting that programming of nutrient sensor signaling in fetal beta-cells as well as the placenta plays a critical role in maintenance of beta-cell health and, subsequently, offspring glucose metabolism.

### Fetal immune system and cytokines

The placenta is the interface between maternal and fetal circulation that modulates signals from maternal immune cells and cytokines during inflammation to produce an integrative response that may be involved in regulating fetal immune function and the production of cytokines. These exert an effect on fetal development. Cytokines are signaling molecules produced by various cells and play a critical role in the regulation of immune function, inflammation, and cell growth and differentiation. Placentae are both targets and producers of a variety of cytokines and chemokines that are critical for appropriate placental development and function, both of which impact the *in utero* placenta–fetal interactions (Szukiewicz, 2012). Various states of maternal inflammation that arise from maternal health status during pregnancy (such as viral infection, maternal obesity, and GDM) can modulate cytokine profiles in the placenta (Zhu *et al.* 2010, Mrizak *et al.* 2014, Elgueta *et al.* 2022). Placentae may be targeted by maternal inflammatory cytokines such as TNF-α, which, in turn, impacts the placental inflammatory secretome by increasing the production of cytokines such as GM-CSF, CCL5 and IL-10 (Siwetz *et al.* 2016). While maternal-derived immune cells or cytokines may directly impact placental production of cytokines, the secretome alterations may be secondary to the immune response. For example, a study into malarial infection during pregnancy showed that the infection was directly associated with higher placental IL-10 levels, while certain cytokines such as IFN-γ and IL-5 levels are associated with low birthweight for gestational age independent of viral infection (Chene *et al.* 2014).

Changes to maternal and placental cytokines may directly impact the function and health of the placenta itself. Mechanistically, cytokines such as IL-6 and TNF-α are associated with the positive expression of amino acid transporters (Jones *et al.* 2009), whereas IL-1β is associated with inhibition of insulin-stimulated amino acid transport in the placenta (Aye *et al.* 2013). Cytokines such as TNF may stimulate mTOR complex 1 (mTORC1) activity, leading to altered expression of nutrient transporters (Brett *et al.* 2014). In addition to affecting placental function, it is feasible that the placental-derived cytokines may enter the fetal circulation to modulate beta-cell growth and development *in utero.* However, this has not been explored. A number of studies have suggested that pro-inflammatory cytokines such as IFN-γ and IL-1β lead to beta-cell dedifferentiation and ER stress-mediated cell death in models of type 1 diabetes and T2D (Nordmann *et al.* 2017, Demine *et al.* 2020, Tesi *et al.* 2021), while anti-inflammatory cytokines may confer protection against diabetogenic stressors to preserve beta-cell mass (Russell & Morgan 2014). Islet dysfunction and destruction observed in the nonobese diabetic (NOD) mice are associated with the presence of cytotoxic cytokines (Jorns *et al.* 2014). However, a protein-restricted diet during pregnancy in NOD mice led to improved offspring islet health, which was associated with increased protective IL-4 and decreased pro-inflammatory IFN-γ signaling in female offspring but not in males (Chamson-Reig *et al.* 2009). This suggests maternal health directly impacts the cytokine profile of the offspring, leading to impacts on beta-cell health. While many of these studies in mature, adult islets suggest that pro-inflammatory cytokines lead to beta-cell dysfunction, some studies suggest that TNF-α and IFN-γ may function as growth factors in fetal beta-cells (Tuch *et al.* 1991).

Recent advancements in immunometabolism have highlighted the role of the tissue resident immune cell population. Macrophage infiltration into the islet milieu is associated with diabetes progression and defects in beta-cell function and mass (Ehses *et al.* 2007), but changes to the population and function of resident macrophages and immune cells during pregnancy may also have a profound effect on fetal beta-cell development (Golden & Simmons 2021). Current studies examining how changes in maternal inflammatory status and placental cytokines impact the fetal immune cells are limited. Some studies do suggest that placental inflammation *in utero* may entrain and prime the fetal immune system to increase cytokine production (Apostol *et al.* 2020). Additionally, new findings suggest that there is vertical transfer of the maternal immune population (maternal microchimeric cells) to the offspring to promote immune development (Stelzer *et al.* 2021). It appears that fetal immune development, much like beta-cell development, is also affected by stressors early in life. However, studies seeking to understand the effect of pregnancy complications on fetal macrophages and how they directly impact offspring beta-cell development are very limited. Reduced uterine placental perfusion (RUPP), a model of preeclampsia, in rats leads to lower beta-cell area in the female offspring, which is associated with increased apoptosis and glucose intolerance in adulthood (Akhaphong *et al.* 2018). The reduction in beta-cell area is associated with increased macrophage number in the female RUPP offspring pancreas, as macrophage depletion via clodronate treatment in RUPP rescues the deficit in beta-cell area (Root *et al.* 2022). Additionally, islets from offspring of diet induced GDM had increased expression of inflammatory genes (Ccl2 and IL-1β) (Agarwal *et al.* 2019). Therefore, inflammatory programming of the offspring immune cell population and/or the islet cells directly may involve cross talk between maternal, placental, and fetal circulation to modulate beta-cell health and development. Further characterization of the relationship between placental cytokines and the fetal immune (both global and islet resident) cell population is required to elucidate the direct role of placental inflammation and islet development.

### MicroRNAs/exosomes

Exosomes are small vesicles that are released by cells and contain a variety of biologically active molecules, including RNA, proteins, and lipids, to impact distal tissue function, growth, and development. These exosomes can be produced by the placental trophoblasts during pregnancy and secreted into the fetal circulation (Luo *et al.* 2009, Record 2014, Mitchell *et al.* 2015). During a healthy pregnancy, the amount of secreted exosome and microparticles by the placenta increases with gestational age and placental size, and the levels can be altered in pregnancy complications such as preeclampsia (Redman & Sargent 2008, Salomon *et al.* 2014). Although current evidence for placental derived exosomes and miRNAs impacting fetal tissues is limited (Xu *et al.* 2021), it is possible for alterations to the levels or content of the exosomes, brought on by the changes to maternal milieu, to directly impact pancreatic beta-cell development and function.

An increasing body of evidence suggests that noncoding RNAs are critical modulators of beta-cell proliferation and function (Sisino *et al.* 2017). Specifically, miRNAs seem to play an important role in regulating the expressions of genes that regulate beta-cell maturation, proliferation, and function (Filios & Shalev 2015). Islet miRNA expression has been shown to be sensitive to prenatal conditions and be important in offspring beta-cell identity and function. For example, miR-7, which is one of the major islet miRNAs shown to regulate beta-cell function and mass through nutrient sensor expression such as mTOR and O-GlcNAc transferase (OGT), has been shown to increase during development (Joglekar *et al.* 2009, Wang *et al.* 2013). Fetal islet miRNA expression patterns are altered in maternal nutrient deprivation conditions where a low protein diet during pregnancy led to the increased expression of miRNAs (199a-3p, -342 and Mir 7), which were associated with poor glucose stimulated insulin secretion in offspring islets (Alejandro *et al.* 2014*b*). In a separate study, maternal low protein diet led to increased expression of miR-15b, which was associated with reduced beta-cell mass and proliferation (Su *et al.* 2016). Consistently, deletion of* Dicer*, one of the genes responsible for miRNA processing, leads to defects in insulin secretion and glucose intolerance in mice, highlighting that endogenous miRNA play an important role in beta-cell mass and function (Kalis *et al.* 2011). However, current data on the impact of exogenous miRNA transport to beta-cells is lacking. Considering the importance of miRNAs during prenatal conditions and that alternations to miRNA profiles in offspring exposed to a poor maternal milieu are associated with defects in beta-cell mass and function, one can speculate that changes in miRNA released by the placenta may play a role in shaping the beta-cell miRNA landscape and thus their action. For example, GDM leads to reduced miRNA, such as miR-148a-3p and miR29a-3p, in the circulating fetal serum (Shah *et al.* 2021). Interestingly, miR-148a has been shown to promote insulin expression, playing a role in beta-cell maturity and identity (Filios & Shalev 2015, LaPierre & Stoffel 2017). It is important to note, however, that these studies are performed postnatally and cannot provide conclusive evidence for placental-derived miRNAs.

### Placental secretagogues

The placenta is an endocrine organ that produces and secretes a myriad of hormones and cytokines to support the growth and development of the placenta and the fetus, including the pancreatic beta-cells. The placental secretome is altered in pathophysiological conditions and are studied in the context of the maternal circulation (Napso *et al.* 2021). Many current studies lack direct evidence of placental hormones secreted into fetal circulation acting on fetal beta-cells during development.

Hormones such as placental lactogen are exclusively produced and secreted by the placenta during pregnancy to modulate the metabolic health of both the mother and fetus (Brelje *et al.* 1993). Placental lactogen is detected in both maternal and fetal circulation after 6 weeks into pregnancy (Handwerger & Freemark 2000, Freemark 2010, Mohan *et al.* 2018). Placental lactogen is thought to play a role in the metabolic reprogramming such as beta-cell expansion of the mothers during pregnancy (Sibiak *et al.* 2020). The prolactin receptor (PRL) is expressed in the beta-cells and is important for maternal beta-cell expansion during pregnancy, leading to increased insulin secretion (Banerjee *et al.* 2016). In the rat pancreas, prolactin receptor (PRLR), a cognate receptor for placental lactogen, is described to be predominantly expressed in islets, particularly in beta- and alpha-cells in late gestation, suggesting potential role of direct placental lactogen on fetal islet development (Freemark *et al.* 1997). Additionally, maternal lactogen levels are positively correlated with IGF-1 and IGF-2 levels in fetal serum, which may, in turn, affect the function and development of the placenta to ultimately impact fetal metabolic programming (Sibiak *et al.* 2020).

Hormones such as leptin, which are normally produced by adipose tissues in the body, are also synthesized and secreted by the placenta. Placental leptin is a hormone that is secreted by the placenta during pregnancy. It plays a vital role in regulating fetal growth and development by controlling energy balance and metabolic function during pregnancy. Leptin works through the leptin receptor activating Jak/STAT pathway to enhance energy mobilization and antagonizes the insulin receptor signaling pathway (Park & Ahima 2014). Placental leptin levels have been shown to be associated with fetal growth and birth weight, with higher levels often linked to larger babies (Valleau & Sullivan 2014). Although the hormone is synthesized and secreted by the syncytiotrophoblast cells in the placenta, the percentage of placental leptin that is secreted into fetal circulation is low (Linnemann *et al.* 2000). It is important also to note that leptin is not secreted from the placenta of all mammals, and its secretion are species-specific traits (Zhao *et al.* 2003). While it is unknown how placental or fetal leptin acts on beta-cell development *in utero,* fetal pancreatic islets express leptin receptor, and leptin is shown to stimulate fetal islet cell proliferation *in vitro* (Islam *et al.* 2000).

While some of the endocrine functions of the placenta have been identified, the majority previous studies have only tested these hormones in the maternal circulation. Newer studies have begun to focus on placental-derived hormones and cytokines that are secreted into the fetal circulation to directly impact fetal tissue development. In one study, utilizing slow off-rate modified aptamer protein binding technology on serum from the maternal radial artery, uterine vein, and the umbilical artery and vein, 1310 known proteins were detected and quantified. A total of 341 proteins were secreted by placenta into fetal circulation, with only 7 distinct proteins being secreted into both maternal and fetal circulation, suggesting discrete directionality of the secretome (Michelsen *et al.* 2019). A different study on the placental secretome from primary human trophoblasts identified 1344 proteins with significant overlap between the *in vitro* secretome and the proteins found in fetal circulation. Upon performing ingenuity pathway analysis, canonical pathways by the secretome showed convergence on mTOR signaling, highlighting the significance of mTOR as a major determinant of organ development (Rosario *et al.* 2021). Other studies also highlight proteins such as superoxide dismutase 3 (SOD3) can be secreted by the placenta, stimulated by maternal exercise, to impact epigenome of the fetal offspring liver and improve glucose tolerance in adulthood (Kusuyama *et al.* 2021).

The identification of secretion of hormones/ligands from the placenta is indeed important. However, without understanding the receptor expression patterns across fetal tissue, the secretome alone cannot elucidate how fetal tissue development may be impacted. For example, a study has associated the expression of placental G-protein coupled receptor (GPCR) ligands with maternal beta-cell GPCR to describe a potential route of interaction between the placenta and fetal islets (Drynda *et al.* 2018). Moving forward, similar approaches on the identifying placental secretome and fetal beta-cell receptors in the context of pregnancy complications are needed to fully understand the adaptation and development of beta-cells *in utero* and their role in the progression of diabetes as adults.

### Considerations for sex and extrauterine maternal impact on beta-cell development

Considering sex as a critical biological variable in metabolic studies is essential, as evident from the distinct responses to pathophysiology that manifest across various diseases. Therefore, it becomes equally important when investigating the prenatal development of both the placenta and the fetus. Indeed, multiple research studies have highlighted differences in the proteome, transcriptome, and metabolome of male and female placentae in both humans and mice. These differences underscore a divergence in terms of immunological and metabolic adaptation (Saoi *et al.* 2020, Braun *et al.* 2022, Olney *et al.* 2022, Baines & West 2023, Maxwell *et al.* 2023). Possible mechanisms that may explain these sex differences include X-inactivation escape of genes, imprinting, and fetal androgen production (Howerton *et al.* 2013, Gong *et al.* 2018, Braun *et al.* 2021, Arevalo *et al.* 2021). In line with this idea, manipulations of placental genes or maternal diet also lead to sex dimorphic responses in offspring glucose metabolism (Yokomizo *et al.* 2014, Akhaphong *et al.* 2018, 2021*a*, Chung *et al.* 2022).

Additionally, it is crucial to recognize that beta-cell development continues after birth. The nourishing support provided to the offspring through lactation might also play a significant role in governing the growth and performance of beta-cells during this period. Maternal milk contains macronutrients, antibodies, vitamins, exosomes (e.g. containing miRNA) hormone (e.g. insulin), and immune cells (e.g. macrophage) (Ballard & Morrow 2013, Cansever *et al.* 2023), These milk components could influence the pancreas development of growing neonates. Therefore, when exploring pregnancy-related complications like GDM and obesity, it is essential to account for the impact of lactation. GDM, for example, brings about changes in milk composition, such as increased triglycerides and alterations in cytokine/chemokine levels (Wu *et al.* 2021, Avellar *et al.* 2022, Dugas *et al.* 2023). Human milk with from women with gestational hypertension show higher composition of fat and carbohydrates in comparison to healthy women (Dangat *et al.* 2016, Sokolowska *et al.* 2023). During the preweaning and weaning phases period in mice, the pancreatic beta-cells enter a pivotal period marked by functional maturation. This developmental stage is characterized by the enhanced ability of these cells to efficiently utilize glucose as their primary substrate for insulin secretion (Stolovich-Rain *et al.* 2015, Jaafar *et al.* 2019). Hence, it's plausible that the content of milk (like miRNAs and nutrients) plays a vital role in the maturation of beta-cells during this developmental stage (Melnik 2019). Future research endeavors focused on elucidating the components within the milk secretome and their precise effects on beta-cell development, function, and maturation hold immense promise in advancing our understanding of fetal programming in relation to T2D.

## Conclusion

A strong body of evidence suggests that the *in utero* environment plays a significant role in the development and function of pancreatic beta-cells. It is being recognized that early life may be a targetable window of time to halt the epidemic of T2D. While studies suggest that flux of nutrients through the placenta and various placental secretagogues (hormones, cytokines, exosomes, etc.) may contribute to the development of beta-cell dysfunction and increase susceptibility to diabetes, further research is needed to fully understand the mechanisms at play. It is clear, however, that the health of the placenta plays a crucial role in fetal development and can have significant implications for the health of the offspring in the long term (Fig. 2 and Table 1). For example, new experiments identifying the placental secretome reaching the pancreas will be very insightful and those demonstrating causality relationships will be essential to define mechanisms of how placental function alters beta-cell function and maturation. Moreover, more experimental efforts to understand the specific factors that contribute to placental inflammation resulting in fetal programming of T2D may be of particular interest. Inflammation is one of the common themes downstream of various pregnancy complications as FGR, GDM, obesity and parity, and to develop interventions to reduce its impact are therefore of great importance in halting the epidemic of obesity and T2D.

**Table 1:** A brief summary of pregnancy complication associated offspring phenotype and possible mechanisms mediated via the placenta.

Pregnancy complications	Offspring outcome	Possible mechanisms
Maternal nutrition and obesity	Undernutrition Human -Increased risk of T2D and obesity Rodent -Glucose intolerance -Reduced beta cell mass Overnutrition/Obesity Human -Increased risk of T2D and obesity Rodent -Altered islet morphology and beta-cell mass/function	Under-nutrition -Reduced nutrient flux -Poor placental development -Altered fetal microRNA landscape Overnutrition/obesity -Excess nutrient flux -Maternal/placental inflammation
Fetal growth restriction (FGR)	Human -Increased risk for insulin resistance and diabetes Rodent -In adulthood, glucose intolerance and obesity -Islet morphological abnormality -Reduced beta cell area, associated with increased cell death	-Reduced nutrient flux -Altered fetal immune cell function
Gestational diabetes (GDM)	Human -Increased weight gain -Impaired glucose tolerance and reduced beta-cell function Rodent -In early life, increased neonatal islet mass and increased insulin levels -In adulthood, reduced *in vivo* and *ex vivo* insulin secretion, and glucose intolerance	-Increased nutrient (glucose) flux -Maternal/placental inflammation -Altered placental secretome (e.g. microRNA)
Multiparity	Human -In early life, improved cardiometabolic health and less adverse neonatal outcomes Nonhuman primate -Glucose intolerance at 3 years of age Rodent -In adulthood, increased obesity in male offspring	-Maternal/placental inflammation -Placental insulin signaling

By addressing the underlying causes of beta-cell dysfunction, it may be possible to reduce T2D incidence and improve the lives of millions of people around the world who live with this chronic condition. Overall, the study of placental and beta-cell development axis represents an exciting new area of research with the potential to improve our understanding of fetal development and health. Continued efforts by the research community and support by various agencies worldwide to investigate this complex relationship will be essential for making progress in the prevention and treatment of diabetes.

## Declaration of interest

The authors declare that there is no conflict of interest that could be perceived as prejudicing the impartiality of this review.

## Funding

Funding supporting this work includes 1R01DK136237, R01DK115720, R21HD100840, and R56DK136293 (to EUA) and F31DK131860 (to SJ).
